# Quality of life of hypertensive men with erectile dysfunction in a tertiary health centre in southern Nigeria

**DOI:** 10.4102/phcfm.v15i1.3875

**Published:** 2023-04-26

**Authors:** Silvia I. Ezemenahi, Anthonia N. Alabi, Oluwagbenga Ogunfowokan, Chibundo U. Nwaneli, Adesuwa Q. Aigbokhaode, Patricia Eseigbe

**Affiliations:** 1 Department of Family Medicine, Faculty of Medicine, Nnamdi Azikiwe University, Awka, Nigeria; 2 Department of Family Medicine, Faculty of Medicine, Nnamdi Azikiwe University Teaching Hospital, Nnewi, Nigeria; 3 Department of Family Medicine, Faculty of Medicine, University of Ilorin, Ilorin, Nigeria; 4 Department of Family Medicine, Faculty of Medicine, Lincoln University, Petaling Jaya Selangor, Malaysia; 5 Department of Internal Medicine, Faculty of Medicine, Nnamdi Azikiwe University, Awka, Nigeria; 6 Department of Medicine, Faculty of Medicine, Nnamdi Azikiwe University Teaching Hospital, Nnewi, Nigeria; 7 Department of Public Health, Faculty of Medicine, Federal Medical Centre, Asaba, Nigeria; 8 Department of Family Medicine, Faculty of Medicine, Bingham University, Karu, Nigeria

**Keywords:** hypertensive men, quality of life (QoL), tertiary healthcare, erectile dysfunction (ED), Nigeria

## Abstract

**Background:**

Erectile dysfunction (ED) is the most common disorder of sexual health seen in men in community studies. A man’s sexual health has been found to be a key factor in determining the capacity for maintaining a healthy relationship.

**Aim:**

This study sought to determine the quality of life of hypertensive men with ED attending the out-patient clinics of Federal Medical Centre (FMC), Asaba, South-South, Nigeria.

**Setting:**

This study was conducted in the Out Patients Clinics (OPC) of FMC, Asaba, Delta state, Nigeria.

**Methods:**

After obtaining approval from the ethics and research committees in Asaba, 184 consenting hypertensive men who met the eligibility criteria were selected by systematic random sampling to participate in the study from October 2015 to January 2016. This study was a cross-sectional survey. Data were collected with a semi-structured interviewer-administered questionnaire adopted from the international index of Sexual Health Inventory for Men (SHIM) and the World Health Organization Quality of Life Scale (WHOQOL-BREF). The study complied with the principles of Helsinki and Good Clinical Practice.

**Results:**

The results showed the mean score for physical domain (58.78 ± 24.37), the psychological domain (62.68 ± 25.93), the social domain (50.47 ± 29.09), and the environmental domain (62.25 ± 18.52). Over a fifth, 11 (22.0%), of the respondents with severe ED had poor quality of life.

**Conclusion:**

This study showed that ED is common in hypertensive men and their quality of life was more impaired than those with normal erectile function.

**Contribution:**

This study contributes to holistic approaches to patient care.

## Introduction

Erectile dysfunction (ED) is the most common disorder of sexual health seen in men.^1^ Although most men feel uncomfortable discussing sex, studies have shown that ED affects their self-esteem and relationships with sexual partners, family and colleagues, and has a huge impact on their quality of life (QoL).^2,3^ Globally, current studies have reported that about 47% – 68% of men with hypertension have some degree of ED,^4,5,6^ and higher numbers of hypertension-related ED exist that tend to be more severe than that found in the general public.^7^ As ED is one of the complications of hypertension, the evaluation of QoL is required in the management of these patients to individualise their care.^8,9^

Two important studies, namely the Massachusetts Male Ageing Study (MMAS) in the United States and the European Male Ageing Study (EMAS), highlighted the concern of ED.^10,11^ Hypertension and its management are contributors to the high prevalence of up to 71% of ED in men.^12,13^ In Qatar, researchers found ED to have a prevalence of 66.2% in hypertensive men; in Thailand the level was 56.2%, in Cameroon 50.6% and in Nigeria 77.2%.^14,15,16,17^ Several hypotheses try to explain the pathophysiology of ED in hypertensive individuals since the pioneering work of Jeremy et al.^18^ who explained the role of endothelial oxidative stress on the genesis of ED; another publication reinforced the finding and even demonstrated that anti-oxidation therapy could improve endothelial function with positive interference on erectile function.^19^ A number of studies have demonstrated the relationship between ED and hypertension.^20,21,22,23,24^

Recently, it has become important to determine QoL, especially as it relates to the health of individuals suffering from chronic illnesses.^9^ One of the methods of comprehensive evaluation of patient health status is QoL assessment and in the management of hypertension, evaluation of QoL helps in individualisation of care for the patients.^9^

Sexual function is one of the important indices of QoL and it promotes psychological well-being as well as interpersonal relationships.^25^ The World Health Organization (WHO) defines sexual health as a continuum of physical, psychological, and socio-cultural well-being associated with sexuality.^26^ It is not merely the absence of disease, dysfunction or infirmity.^27^

So monitoring the indices of QoL in hypertensive men with ED is useful to boost their confidence and self-esteem for a better QoL.^25^ Quality of life according to the WHO is defined as ‘an individual’s perception of their position in life in the context of the culture and value systems in which they live and in relation to their goals, expectations, standards and concerns’.^28^ It is affected by the person’s physical health, personal beliefs, psychological state, social relationships and their relationship to salient features of their environment.^28^

As early as 1982, Jachuck et al.^29^ reported an association between sexual dysfunction and impairment of QoL in hypertensive patients treated primarily with diuretics, beta blockers, or methyldopa.^29^ Since then, other studies have reported that ED has an effect on the QoL of men.^25,30^ A study showed that men who suffer from ED will have poor QoL, especially in physical and social domains.^31^ Most of the medical treatments for ED now focus on improving QoL of patients as QoL is used to assess the overall well-being of a person.^2^

Some researchers were able to demonstrate that it was difficult to assess the real effect of ED on QoL because ED could be due to psychological or other medical conditions.^32,33^ while others reported that men with ED and comorbid medical conditions have poorer QoL as they are more distressed and less satisfied with their sexual life.^34,35^

Determination of QoL especially as it relates to the health of individuals suffering from chronic illnesses is important but considering that QoL is difficult to define in universally accepted terms let alone to quantify, to date there is no one survey tool that has been deemed the gold standard to measure QoL in patients.^36^

The WHO in 1996 developed a QoL assessment tool due to a need for a genuinely international measure of QoL, and as a commitment to the continued promotion of a holistic approach to healthcare.^37^ Together with other measures, the World Health Organization Quality of Life Scale (WHOQOL-BREF) will enable health professionals to assess changes in QoL over the course of treatment.^37^ A review of literature shows there is a paucity of studies reporting QoL of hypertensive men with ED. To our knowledge no study has been done to determine the QoL of hypertensive men already living with ED in Nigeria. Current data on QoL among hypertensive men with ED, particularly for physiological and psychosocial variables, are extremely lacking in Nigeria despite the prevalence and implications of ED on QoL of healthy populations.^38^

The aim of the study was to determine the QoL of men living with hypertension and ED attending outpatient clinics of the Federal Medical Centre (FMC) in Asaba, Nigeria.

## Research methods and design

This was a descriptive cross-sectional study carried out among 184 hypertensive men who were bilingual in English and pidgin English and aged 18 years and above. Participants were recruited from the outpatient clinics of FMC, Asaba, Nigeria, using systematic random sampling, from October 2015 to January 2016.

### Setting

The study was conducted in the outpatient clinics of the FMC, Asaba, Nigeria, which comprises the children, the adult and the geriatrics clinics. The clinics are run by family physicians. This study was conducted in the adult and the geriatrics arms of the clinics. The FMC is the only federal tertiary health institution in Delta state, Nigeria. Asaba is an ancient city located in Oshimili south local government area, north senatorial zone of Delta state, Nigeria. It sits on the bank of the river Niger overlooking the south-eastern commercial city of Onitsha. It is the capital of Delta state with an estimated population of 149 603.^39^

### Study population

The study population consists of all male hypertensive patients on follow-up at outpatient clinics of the FMC. Approximately 250 male hypertensive patients are seen each month in the clinics (from data in medical records). For the 3 months of the study, a total of 750 hypertensive males were seen, out of whom 184 participants were recruited. The first patient was selected by balloting within the sampling interval after which every consecutive fourth patient was recruited from the population of 750 patients until the sample size of 184 was drawn. Eligibility criteria were: male hypertensive patients aged 18 years or above and engaged in sexual activity over the past 6 months prior to data collection, regardless of the duration of hypertension, history of smoking, and alcohol consumption status, but without a history of a surgical condition that could cause ED.

### Sample size determination

The sample size was calculated using the formula for studying proportions with populations greater than 10 000.^40^

Since we are dealing with unknown prevalence, we used the prevalence from a published study on hypertensive men with poor QoL conducted at a Lagos university teaching hospital, Nigeria, by Mbakwem et al.^41^ The study found overall poor QoL prevalence to be 12.2% in hypertensive men:


$$n=\frac{z^{2}pq}{d^{2}}$$ [Eqn 1]

*d* = precision; 5% from the population proportion was selected.

*z* = confidence limits of the survey result; 95% (z = 1.96) was chosen.

This means that a range of ±10% from the sample result should include the true result in 95 of every 100 surveys performed. After adding a 10% nonresponse rate, the total sample size was 184.

### Data collection

The procedure for the study was explained to each participant and thereafter informed consent was obtained before their participation in the study. Information was obtained through interview and the interviewers were mainly the principal investigator and a nurse from FMC who was trained to administer and complete the questionnaires. To enhance accuracy, all participants were informed that their responses would remain confidential. The questionnaire was divided into five sections: socio-demographic details, medical history, social history, degree of ED, assessed with the international index of Sexual Health Inventory for Men (SHIM),^42^ and QoL, assessed using the WHOQOL-BREF^37^ questionnaire.

The SHIM was an abridged 5-item version of the 15-item International Index of Erectile Function (IIEF-15). The SHIM is an already validated tool used to detect ED and assess its severity. A respondent scores 1–5 points on each of five questions about his erectile function. The SHIM score is the sum of the individual question scores. The lowest total score was 5 and the highest total score was 25. Erectile dysfunction was classified into levels: (1) severe = 1–7; (2) moderate = 8–11; (3) mild-to-moderate = 12–16; (4) mild = 17–21; (5) normal erectile function = 22–25.

For QoL the WHOQOL-BREF questionnaire was used; it contains two items from the Overall QoL and General Health and 24 items of satisfaction that were divided into four domains: physical health with seven items (DOM1), psychological health with six items (DOM2), social relationships with three items (DOM3) and environmental health with eight items (DOM4). The two items are examined separately: question one asked about an individual’s overall perception of QoL and question two asked about an individual’s overall perception of their health. Each item was rated on 5-point Likert scale. Each item of the WHOQOL-BREF was scored from 1 to 5 on a response scale. Raw domain scores for the WHOQOL-BREF were transformed to a 4–20 score according to guidelines. Domain scores were scaled in a positive direction (i.e. higher scores denote higher QoL). The mean score of items within each domain is used to calculate the domain score. Once computed, the scores were transformed linearly to a 0–100-scale.

### Data analysis

Data were checked for completeness, then entered and analysed using Statistical Package for Social Science (SPSS) software, version 20.0 (IBM Inc., Armonk, New York, United States). Descriptive analyses performed including frequencies, percentages, means, and standard deviations (s.d.) and Chi-square was used to test associations. Pearson’s correlation coefficient was used to determine the level of agreement between four domains of the WHOQOL-BREF. Transformed scores were used for statistical analyses in the four domains. In this study, the level of significance was set at *P* < 0.05 for all analyses.

### Ethical clearance

Ethical clearance for the study was obtained from the Federal Medical Centre Ethics and Research committee. Written informed consent was obtained in the common spoken language (English, Ibo or pidgin English) from each participant. All information obtained from subjects was treated confidentially. Respondents identified as having other previously undiagnosed medical conditions were followed up with appropriate intervention. Cost of materials and investigations were borne by the researchers without any cost to the participants.

## Results

Of the 750 eligible individuals (adult male patients with hypertension) over the study period, 184 participants (24.5%) were included, all of whom participated without any dissent. Fifty-one (27.7%) of the respondents were in the age group of 40–49 years followed by 46 (25.0%) in the 50–59-year age group with 3 (1.6%) in the age group of 80–89 years with a mean age (±s.d.) of the respondents being 55.1 (±12.4) years. Sixty-two (33.7%) of the respondents had university education, followed by 44 (23.9%) with post-secondary qualifications, while 35 (19.0%) had secondary education. Three-quarters of the respondents (*n* = 138) were married, 15 (8.2%) were single, 7 (3.8%) were cohabiting and 16 (8.7%) were widowed, as seen in Table 1.

**TABLE 1:** Socio-demographic characteristics of respondents.

Variable	Frequency (*N* = 184)	%
**Age group (years)** †		
30–39	17	9.3
40–49	51	27.7
50–59	46	25.0
60–69	41	22.3
70–79	26	14.1
80–89	3	1.6
**Level of education**		
None	11	6.0
Primary	32	17.4
Secondary	35	19.0
Post-secondary	44	23.9
University	62	33.7
**Marital status**		
Single	15	8.2
Married	138	75.0
Divorced	8	4.3
Cohabiting	7	3.8
Widowed	16	8.7

† , Mean age 55.1 (± 12.4).

Fewer than a fifth (27; 19.01%) of the respondents with ED had very good QoL. More than half of the respondents without ED had very good QoL, as shown in Table 2.

**TABLE 2:** Quality of life of respondents with and without erectile dysfunction.

Quality of life	Frequency	%	95% confidence interval
**Respondents with erectile dysfunction (*N* = 142)**			
Very poor	14	9.86	5.96 – 15.87
Poor	28	19.72	14.01 – 27.02
Neither poor nor good	40	28.17	21.42 – 36.07
Good	33	23.24	17.05 – 30.84
Very good	27	19.01	13.41 – 26.25
**Respondents without erectile dysfunction (*N* = 42)**			
Very poor	3	7.1	24.6 – 19.01
Poor	0	0	0.00 – 8.38
Neither poor nor good	3	7.1	2.46 – 19.01
Good	11	26.3	15.3 – 41.07
Very good	25	59.5	44.49 – 72.95

Forty-four (23.9%) of the respondents were satisfied with their health status, 45 (24.5%) were dissatisfied and 16 (8.7%) were very dissatisfied, as shown in Table 3.

**TABLE 3:** Health status satisfaction among respondents.

Health status satisfaction	Frequency (*N* = 184)	%	95% confidence interval
Very dissatisfied	16	8.7	5.4 – 13.7
Dissatisfied	45	24.5	18.8 – 31.1
Neither satisfied nor dissatisfied	50	27.2	21.3 – 34.0
Satisfied	44	23.9	18.3 – 30.6
Very satisfied	29	15.7	11.2 – 21.7

Transformed scores for the main domains, physical health, psychological health, social relationships and environment domains, were 58.7826 ± 24.3691, 62.6793 ± 25.9257, 50.4674 ± 29.0909 and 62.2446 ± 18.5234 (Table 4).

**TABLE 4:** Test data set descriptive statistics for the different domains.

Variables	Frequency (*N*)	Minimum	Maximum	Mean	Standard deviation
Physical (Transformed)	184	0.0	100.0	58.7826	24.3691
Psychological (Transformed)	184	6.0	100.0	62.6793	25.9257
Social relationships (Transformed)	184	0.0	100.0	50.4674	29.0909
Environment (Transformed)	184	19.0	100.0	65.2446	18.5234

A weak positive correlate (*r* = 0.417) was found between perceived QoL and health status and the physical domain. This, however, was statistically significant (*P* < 0.001). There was a strong correlation between the psychological domain and perceived quality of health and health status of the respondents and this was statistically significant (*r* = 0.868, *P* < 0.001). A strong positive correlation (*r* = 0.698) was found between perceived QoL and health status and the social relationships domain. This was statistically significant (*P* < 0.001). There was a strong correlation between the environment domain and perceived quality of health and health status of the respondents and this was statistically significant (*r* = 0.730, *P* < 0.001) (Table 5).

**TABLE 5:** Pearson’s correlation of quality of life and health status with the four domains among respondents.

Variables	Perceived quality of life and health status	Domains	*P*-value
Perceived quality of life and health status	1	0.417	< 0.001
Physical domain	0.417	1	< 0.001
Perceived quality of life and health status	1	0.868	-
Psychological relationships domain	0.868	1	< 0.001
Perceived quality of life and health status	1	0.698	-
Social relationships domain	0.698	1	-
Perceived quality of life and health status	1	0.730	< 0.001
Environment domain	0.730	1	-

Sixty-six (35.9%), 55 (29.9%), 73 (39.7%) and 29 (15.8%) of the respondents had domain scores less than 50% for physical health, psychological health, social relationships and environment domains, as shown in Table 6.

**TABLE 6:** Domain scores for less than 50% and greater than 50%.

Variables	Frequency < 50%		Frequency ≥ 50%	
	Frequency	%	Frequency	%
Physical (Transformed)	66	35.9	118	64.1
Psychological (Transformed)	55	29.9	129	70.1
Social relationships (Transformed)	73	39.7	111	60.3
Environment (Transformed)	29	15.8	155	84.2
Quality of life and health status (Transformed)	54	29.3	130	70.7

Note: Frequency (*N* = 184).

Using a non-parametric statistical test (Chi-square) to test associations between ED and QoL, it was discovered that a greater proportion (85.8%) of respondents without ED had good QoL compared to those with severe, moderate and mild ED: 26 (52.0%), 13(39.4), 13(59.1); *P* < 0.001, as shown in Table 7.

**TABLE 7:** Association between erectile dysfunction and quality of life of respondents.

Degree of Erectile dysfunction	Quality of life (*n* = 184)							
	Poor		Neither poor nor good		Good		Total	
	Frequency	%	Frequency	%	Frequency	%	Frequency	%
Severe	16	32.0	8	16.0	26	52.0	50	100.0
Moderate	13	39.4	7	21.2	13	39.4	33	100.0
Mild moderate	9	24.3	20	54.1	8	21.6	37	100.0
Mild	4	18.2	5	22.7	13	59.1	22	100.0
Normal	3	7.1	3	7.1	36	85.8	42	100.0

Note: *χ*^2^ = 47.458, *P* < 0.001.

A greater proportion of respondents with normal erectile function (22; 52.3%) were very satisfied with their health status compared to those with severe (2; 4.0%), moderate (1; 3.0%), mild moderate (3; 8.1%) and mild erectile dysfunction (1; 4.5%), as shown in Table 8.

**TABLE 8:** Association between degree of erectile dysfunction and health satisfaction among respondents *χ*^2^ 96.800, *P* < 0.001.

Degree of erectile dysfunction	Health satisfaction									
	Very dissatisfied		Dissatisfied		Neither		Satisfied		Very satisfied	
	Frequency	%	Frequency	%	Frequency	%	Frequency	%	Frequency	%
Severe	4	8.0	21	42.0	12	24.0	11	22.0	2	4.0
Moderate	5	15.2	12	36.4	8	24.2	7	21.2	1	3.0
Mild moderate	3	8.1	9	24.3	20	54.1	2	5.4	3	8.1
Mild	3	13.7	1	4.5	6	27.3	11	50.0	1	4.5
Normal	1	2.4	2	4.8	4	9.5	13	31.0	22	52.3

A greater proportion (32; 64.0%) of respondents with severe erectile dysfunction had average score of < 50% in domain 1, as seen in Table 9.

**TABLE 9:** Association between degree of erectile dysfunction and domains of quality of life.

Degree of erectile dysfunction	Domains of quality of life†							
	Domain 1		Domain 2		Domain 3		Domain 4	
	< 50%	> 50%	< 50%	> 50%	< 50%	> 50%	< 50%	> 50%
Severe ED	32 (64.0)	18 (36.0)	19 (38.0)	31 (62.0)	30 (60.0)	20 (40.0)	11 (22.0)	39 (70.0)
Moderate ED	17 (51.5)	16 (48.5)	14 (42.4)	19 (57.6)	20 (60.6)	13 (39.4)	9 (27.3)	24 (72.7)
Mild moderate ED	12 (32.4)	25 (67.6)	13 (35.1)	24 (64.9)	18 (48.6)	19 (51.4)	7 (18.9)	30 (81.1)
Mild ED	3 (13.6)	19 (86.4)	6 (27.3)	16 (72.7)	3 (13.6)	19 (86.4)	1 (4.5)	21 (95.5)
Normal	2 (4.8)	40 (95.2)	3 (7.1)	39 (92.9)	2 (4.8)	40 (95.2)	1 (2.4)	41 (97.6)

† , average score of 50%.

## Discussion

The study was undertaken to determine the QoL of hypertensive men with ED attending the outpatient clinics at FMC, Asaba, Nigeria.

A total of 17 (9.2%) of the respondents felt their QoL was very poor while 45 (24.5%) were dissatisfied with their health status. Transformed scores for the main domains, physical health, psychological health, social relationships and environmental, were 58.78 (±24.37), 62.68 (±25.93), 50.47 (±29.09) and 65.25 (±18.52). Among the four domains of the WHOQOL-BREF, the highest mean score was observed in the environment domain, implying that the study population had relatively more satisfaction with their financial resources, freedom, physical safety and security. They also had satisfaction with their health and social care: accessibility and quality. In addition, they had satisfaction with their opportunities for acquiring new information, skills, participation in and opportunities for recreation and leisure activities, physical environment (pollution, noise, traffic, climate) transport. Moreover, the lowest mean score was observed in the social relationship domain, in which sexual function belongs, indicating low satisfaction with their personal relationships, sexual activity and social support. These findings are similar to a study done by Odili and colleagues in Benin, Nigeria, using the same methodology but among patients with diabetes the mean score for the psychological health domain was 56.28 (± 14.88).^43^ Similar findings were also reported by Issa and colleagues at Ife, Nigeria.^44^ The finding in this study was dissimilar to those done by Ninh and colleagues in a rural Vietnam community among hypertensive patients.^45^ The QoL among hypertensive patients was found to be moderate in all domains, except for the psychological domain in which it was fairly low (mean = 49.4).

In this study, 35.9%, 29.9%, 39.7% and 15.8% of the respondents had scores less than 50% for physical health, psychological health, social relationships and the environmental domain. As there are no similar studies reported in the literature, this study becomes important in this regard.

Another interesting finding from this study was the varied individual reaction to ED among the men, with ED having different impacts on their QoL. Some men do not seem to be considerably bothered by poor ED, while others exhibit particular concern and anxiety, resulting in impaired QoL. Of the respondents with severe ED, who were more likely to be in the age group of 60 and above, 11 (22.0%) of the respondents felt their QoL was poor, while 26 (52.0%) felt their QoL was good to very good. These findings could be a reflection of two factors: first the sociocultural characteristics of the society where the study was conducted. In this part of the country where sexuality and its dysfunction are still regarded as a taboo subject, most people would not want to be labelled as having such problems or, by extension, be seen as it affecting their QoL. Second, most of the respondents who were above 60 years of age and had completed their family size believed that they did not need sex but want to focus on being alive and managing current medical issues rather than sexual issues and desire. The devastating effect on QoL was more striking when it occurred in younger age groups. Twenty (54.1%) of those with mild moderate ED had neither poor nor good QoL.

### Limitations

This was a hospital-based study; the patients’ characteristics may therefore not be completely representative of the larger population in Asaba and its environs and, thus, the findings may not be completely extrapolated beyond the study population.

## Conclusion

Erectile dysfunction is common in hypertensive men and their QoL was more impaired than those men with normal erectile function. Therefore, promotion of a holistic approach to healthcare is encouraged for hypertensive patients.
